# Thermocatalytic Behavior of TiO_2_ as a Dehydrogenation Catalyst: A Case Study of Methane Activation and Nonoxidative Coupling

**DOI:** 10.1021/acsomega.5c10988

**Published:** 2026-01-20

**Authors:** Juganta K. Roy, Mona Abdelgaid, Giannis Mpourmpakis

**Affiliations:** † Department of Chemical and Petroleum Engineering, 6614 University of Pittsburgh, Pittsburgh, Pennsylvania 15261, United States; ‡ Department of Chemistry and Physics, 14741 West Texas A&M University, Canyon, Texas 79016, United States; § School of Chemical Engineering, National Technical University of Athens (NTUA), Athens GR-015780, Greece

## Abstract

The abundance of cheap natural gas has changed the energy supply landscape and spurred efforts to find alternative sources of energy to traditional fossil fuels. Methane (CH_4_) is the primary constituent of natural gas, and its C–H bond activation remains a long-standing puzzle in the chemical industry. Transition-metal oxides exhibit intrinsic Lewis acid–base properties beneficial for activating the C–H bonds of CH_4_. In this work, we investigated the nonoxidative coupling of CH_4_ (NOCM) to C_2_ hydrocarbons on the rutile TiO_2_ (110) surface at 1240 K by using density functional theory (DFT) calculations. We explored three different CC coupling pathways for the formation of ethane after the sequential activation of two CH_4_ molecules. We found that CH_3_/CH_3_ coupling involves high activation barriers, while the formation of C_2_H_5_ from the coupling of CH_3_/CH_2_ is kinetically and thermodynamically more facile. Considering ethylene formation routes, we found that the dehydrogenation of methyl species requires high energy barriers. However, the subsequent CC coupling of CH_2_/CH_2_ occurs at a lower activation barrier of 1.01 eV. Moreover, our calculations revealed that the dehydrogenation of C_2_H_5_ to form ethylene is favored over its hydrogenation to form ethane. This work provides various mechanistic pathways that can help in designing dehydrogenation catalysts with enhanced catalytic activity. However, our results indicate that despite low barrier coupling routes, rutile TiO_2_ alone is not an effective catalyst for NOCM due to the energy-intensive C–H activation and limited stability of reactive intermediates. Rutile TiO_2_ may have enhanced activity and selectivity in doped configurations or as a catalyst support within multifunctional catalytic systems.

## Graphical abstract

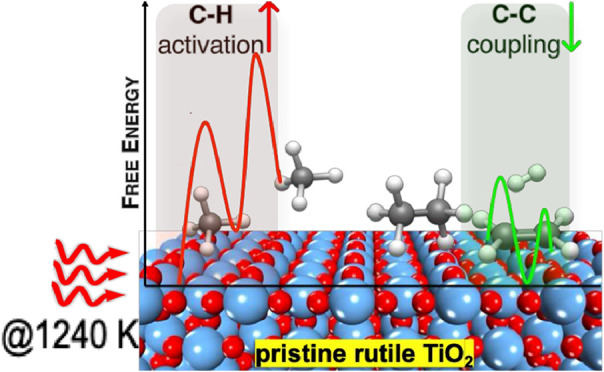

## 1 Introduction

Technological advances in hydraulic fracking and increasing shale gas reserves make natural gas an attractive feedstock for producing valuable chemicals.^1–3^ Methane (CH_4_) is the major constituent of natural gas, with the highest H/C ratio among all hydrocarbons making CH_4_ a valuable fuel source as it burns more efficiently (more energy release from combustion).^4^ CH_4_ is an obvious source of C_2+_ hydrocarbons, carbon monoxide, and hydrogen.^5,6^ However, the conversion of CH_4_ is thermodynamically unfavorable at room temperature as CH_4_ possesses low electron affinity, low polarizability, and high C–H bond energy.^5,7^ Thus, the indirect route of CH_4_ conversion requires high reaction temperature (>700 °C) and pressure (>10 atm) to reform CH_4_ to synthesis gas (CO and H_2_), which can further be converted to higher value-added chemicals like methanol, olefins, formic acid, and aromatics.^6,8,9^

Alternatively, nonoxidative coupling of CH_4_ (NOCM) to C_2+_ hydrocarbons^10^ has been proposed as a more economical and environmentally friendly technology for operation at small capacity owing to the higher process efficiency and reduced number of process steps compared to the multistep indirect processes.^11^ However, NOCM suffers from unfavorable thermodynamic constraints, which necessitate high temperatures above 1200 K.^12–15^ Under harsh reaction conditions, coking of the catalyst is inevitable, hindering the commercialization of the NOCM processes. Therefore, designing an active, selective, and stable catalyst for NOCM is the holy grail in catalysis of the twenty-first century.^16^

In 2014, Bao and colleagues reported a breakthrough in NOCM chemistry using single iron sites embedded in a silica matrix with methane conversion of 48.1%, ethylene selectivity of 48.4%, and total hydrocarbon selectivity exceeding 99% at 1363 K.^12^ To reduce the reaction temperature to 1273 K, Oh et al. designed a millisecond catalytic wall reactor, allowing the reaction to proceed on the reactor wall and within the reactor channel with a resistance time of a few milliseconds.^15^ Kim et al.^14^ performed microkinetic study at 1300 K to get mechanistic insights for the dominant C_2_ products of NOCM on a single-atom iron catalyst. In agreement with the experiments, the authors found that acetylene is the main product of the NOCM reaction at 1300 K.

Metal oxides have drawn enormous attention in CH_4_ activation and conversion due to their inherent Lewis acidic (unsaturated metallic centers) and basic (lattice oxygen centers) properties.^16–24^ Jiang et al. studied CH_4_ adsorption and dissociation on IrO_2_ using density functional theory (DFT) and revealed a lower C–H bond activation energy barrier than CH_4_ desorption energy, introducing IrO_2_ as a potential catalyst for CH_4_ activation.^25^ The Jiang group further proposed a molecular-mediated mechanism for activating CH_4_ on the IrO_2_ (110) surface and converting it to ethylene.^26^ However, the high cost of IrO_2_ hinders its industrial application. Cao and co-workers performed a machine-learning study using surface-structure descriptors to predict the reaction mechanism of CH_4_ activation on rutile oxide surfaces.^27^ Tsuji and Yoshizawa studied the bonding nature of CH_4_/metal and the mechanism of methane C–H bond activation on rutile metal oxides (e.g., CrO_2_, IrO_2_, PtO_2_) using DFT.^28,29^ PtO_2_ was identified as the most active catalyst for CH_4_ activation, outperforming IrO_2_.^28^ However, the industrial application of PtO_2_ as a CH_4_ activation catalyst is limited due to the prohibitively high cost and less abundance of Pt.^30^ Consequently, developing economical and environmentally friendly metal-oxide catalysts for CH_4_ activation and conversation is highly desired.

Among transition-metal oxides, rutile TiO_2_ is one of the most thoroughly studied catalysts, both experimentally and theoretically, and has served as an economical prototypical model for fundamental studies of reducible metal oxides.^31–34^ TiO_2_ has been used as a support to anchor single metals due to its tunable porous surface and high thermal stability. The interactions of TiO_2_ with the metals influence its catalytic activity and selectivity.^34^ Nachimuthu et al. revealed comparable catalytic activity of IrO_2_ supported on TiO_2_ (IrO_2_/TiO_2_) and pristine IrO_2_ toward the NOCM reaction, with CH_4_ activation energies of 0.29 and 0.24 eV, respectively.^35^ Our recent work provides a mechanistic understanding of NOCM on pristine TiO_2_ through multiscale simulations, which reveal the use of light and heat necessary to produce ethane and hydrogen.^36^ Our calculations suggest that photocatalysis is preferred over thermal catalysis for CH_4_ activation (0.30 eV vs 1.01 eV) and CC coupling (0.28 eV vs 2.83 eV), while heat is necessary for catalyst regeneration and production of H_2_. It should be noted that our prior work did not explore the full thermocatalytic pathways for NOCM to ethane and ethylene; rather, it was limited to a comparative analysis of CH_4_ activation and CC coupling under photocatalytic and thermocatalytic conditions. To the best of our knowledge, there is currently a lack of theoretical studies on the thermocatalytic NOCM and possible CC coupling mechanisms on pristine TiO_2_ in the literature.

Further research on NOCM to C_2_ hydrocarbons on TiO_2_ can provide a complete mechanistic understanding of designing new catalysts by revealing the reaction mechanisms and rationales for catalyst deactivation and product selectivity, pushing the current state of the art. Toward this goal, we use first-principles calculations to investigate various ethane (C_2_H_6_) and ethylene (C_2_H_4_) formation mechanisms on TiO_2_. The basic knowledge of CH_4_ activation and conversion on TiO_2_ will provide a good reference point for studying TiO_2_ as a heterogeneous catalyst support or a host material for heteroatoms. Additionally, the mechanistic aspects of the catalytic transformations of methane on TiO_2_ are transferable to other rutile oxides with similar structures to TiO_2_.

## 2 Computational Methods

All plane-wave DFT calculations were performed using the projector augmented wave (PAW)^37^ method, as implemented in the Vienna Ab Initio Simulation Package (VASP 5.4.4).^38,39^ The Perdew–Burke–Ernzerhof (PBE) exchange–correlation functional^40^ was used with a plane-wave cutoff of 400 eV. The van der Waals dispersion forces between the adsorbates and surfaces were considered using Grimme’s DFT-D3 dispersion correction scheme^41^ with Becke–Johnson damping.^42^ The core and valence electrons were modeled by the PAW method with plane-wave basis functions of Ti­(3s^2^3p^6^4s^2^3d^2^), C­(2s^2^2p^2^), O­(2s^2^2p^4^), and H­(1s^1^) pseudopotentials. The Brillouin zone integration was sampled with a **Γ** centered (3 × 2 × 1) Monkhorst–Pack k-mesh with (3 × 2) supercells and Gaussian smearing (σ = 0.2 eV). All the structures were relaxed until the forces on each ion were less than 0.05 eV/Å with an SCF convergence criterion of 1 × 10^–5^ eV. A Hubbard onsite Coulomb repulsion term^43^ was added (DFT + U) to improve the description of the onsite Coulomb interaction in the Ti-3*d* states with an effective U (U–J) parameter. Choosing an effective U value is crucial as the band gap of rutile TiO_2_ is directly proportional to the U values. Moreover, an increment in the *U* values overestimates the experimental lattice parameters.^44^ Our benchmarking calculations followed the same trend (Figure S1). Based on the study of German et al.^45^ and our benchmark calculations, we chose an effective U value of 5.0 eV for Ti-3d orbitals. The climbing image nudged elastic band (CI-NEB)^46^ and dimer methods^47^ were used to optimize the transition-state (TS) structures. All TSs have been confirmed with the existence of a single imaginary frequency along the reaction coordinate.^20^

Rutile TiO_2_ was considered in this work due to its high thermal stability,^48^ with optimized unit cell parameters of *a* = *b* = 4.651 Å and *c* = 2.971 Å, in agreement with experimental^31^ parameters (*a* = *b* = 4.593 Å and *c* = 2.958 Å). The most thermodynamically stable (110) TiO_2_ surface was used to build the supercell, which contains equal numbers of five-coordinated (Ti_5c_) and six-coordinated (Ti_6c_) Ti atoms linked with two types of oxygen atoms in two-coordinated (O_2c_) and three-coordinated (O_3c_) coordination. A 3 × 2 (110) TiO_2_ slab of four O–Ti–O layers was modeled. The bottommost two O–Ti–O layers were fixed during geometry optimization, whereas the upper two atomic layers and the adsorbates were relaxed in all structural optimizations. Neighboring slabs were separated by at least an 18 Å vacuum. The adsorption energy of a gas-phase molecule is defined as *E*_ads_ = *E*_sur+ads_ – *E*_sur_ – *E*_ads_, where *E*_sur+ads_, *E*_sur_, and *E*_ads_ are the total energy of the adsorbate on the surface, a clean surface, and an isolated adsorbate molecule in the gas phase, respectively. The reaction energy was calculated as the energy difference between the initial state (IS) and the corresponding final state (FS), where Δ*E* = *E*_FS_ – *E*_IS_. The activation energy (*E*_a_) was determined as the energy difference between the TS and the corresponding IS, *E*_a_ = *E*_TS_ – *E*_IS_. The Gibbs free energies (*G*) of all states in the reaction energy profiles were calculated using statistical thermodynamics as per the formula *G* = *E* + ZPE + ∫*C*_p_d*T* – *TS*, where *E* is the electronic energy of each system, ZPE is the zero-point energy, *C*_p_ is the heat capacity from 0 to 1240 K, *T* is the temperature, and *S* is the entropy. Several studies have reported NOCM on different types of catalysts at temperatures ranging between 1200 and 1300 K.^12–15^ Based on these studies, we used 1240 K to obtain NOCM free energy profiles. The vibrational modes of only the adsorbates were factored into free energy calculations. Gas-phase corrections to CH_4_, C_2_H_6_, C_2_H_4_, and H_2_ were calculated in the ideal gas approximation, whereas free energy corrections to adsorbed and transition states were computed in the harmonic oscillator approximation.^49^

## 3 Results and Discussion

### 3.1 Dissociative Activation of CH_4_ on Rutile (110) TiO_2_ Surface

#### 3.1.1 Active Sites for CH_4_ Activation

We first investigated the adsorption of CH_4_ on four distinctive metal and oxygen sites of the rutile TiO_2_ (110) surface, namely, Ti_5c_, Ti_6c_, O_2c_, and O_3c_, as shown in Figure 1a. We noticed that the adsorption of CH_4_ is weakly exothermic, with adsorption energies ranging from −0.02 to −0.30 eV (Figure 1b and Table S1). Fung et al. reported CH_4_ adsorption energy on rutile IrO_2_ (110) surface of −0.36 eV,^50^ aligning with our DFT calculations. The optimized bond lengths between the C atom of the adsorbed CH_4_ and Ti_5c_, Ti_6c_, O_2c_, and O_3c_ adsorption sites are 2.99, 4.06, 3.55, and 3.28 Å, respectively, indicating a typical physisorption. In general, two potential surface mechanisms exist for C–H bond activation of CH_4_ on oxide catalysts: the polar and radical pathways.^16,18,51^ The former mechanism is characterized by polar (heterolytic) dissociation of CH_4_ to form CH_3_^–*^ and surface-adsorbed H^+*^. In contrast, the radical pathway is characterized by homolytic dissociation of CH_4_ through the abstraction of a hydrogen radical by a surface oxygen atom, leaving behind a methyl radical in the gas phase.^21^ To examine the C–H bond activation of methane, we selected different distinctive site pairs on the TiO_2_ (110) surface (Figure S2). Specifically, the selected site pairs are ^C^Ti_5c_–^H^O_2c_, ^C^O_2c_–^H^Ti_5c_, ^C^Ti_6c_–^H^O_2c_, ^C^O_2c_–^H^Ti_6c_, ^C^Ti_5c_–^H^O_3c_, ^C^O_3c_–^H^Ti_5c_, ^C^Ti_6c_–^H^O_3c_, and ^C^O_3c_–^H^Ti_6c_, where superscripts C and H indicate adsorption of CH_3_^*^ and H* species on specific sites, respectively. Our DFT calculations found that CH_4_ activation occurs through heterolytic dissociation on the rutile TiO_2_ (110) surface. All attempts to locate the TS of the radical pathway on different active sites failed. The lowest C–H bond activation barrier (1.01 eV) was obtained on the ^C^Ti_5c_–^H^O_2c_ site pair, followed by the ^C^Ti_5c_–^H^O_3c_ site pair (1.64 eV), demonstrating the strong acidity of Ti atoms with a low surface coordination number. Adsorption of CH_3_^*^ species on the O atoms exhibited high energy barriers of 3.32 and 3.86 eV for sites ^C^O_2c_–^H^Ti_5c_ and ^C^O_3c_–^H^Ti_5c_, respectively. It is important to note that all attempts to localize the TS for CH_4_ dissociation on ^C^Ti_6c_ have failed due to the weak adsorption of CH_4_.^18,50^ The reaction energy of CH_4_ dissociation on all site pairs was found to be endothermic. Having observed the relatively preferential adsorption energy (−0.30 eV) and low C–H bond activation energy barrier (1.01 eV) on ^C^Ti_5c_–^H^O_2c_, we investigated NOCM to C_2_ hydrocarbons (C_2_H_6_ and C_2_H_4_) at 1240 K^12–15^ on the most active site pair ^C^Ti_5c_–^H^O_2c_. DFT study by Zhou et al. found CH_4_ adsorption energy of −0.32 eV and C–H bond activation energy barrier of 1.12 eV on pristine TiO_2_, in great agreement with our results.^23^

**Figure 1:**
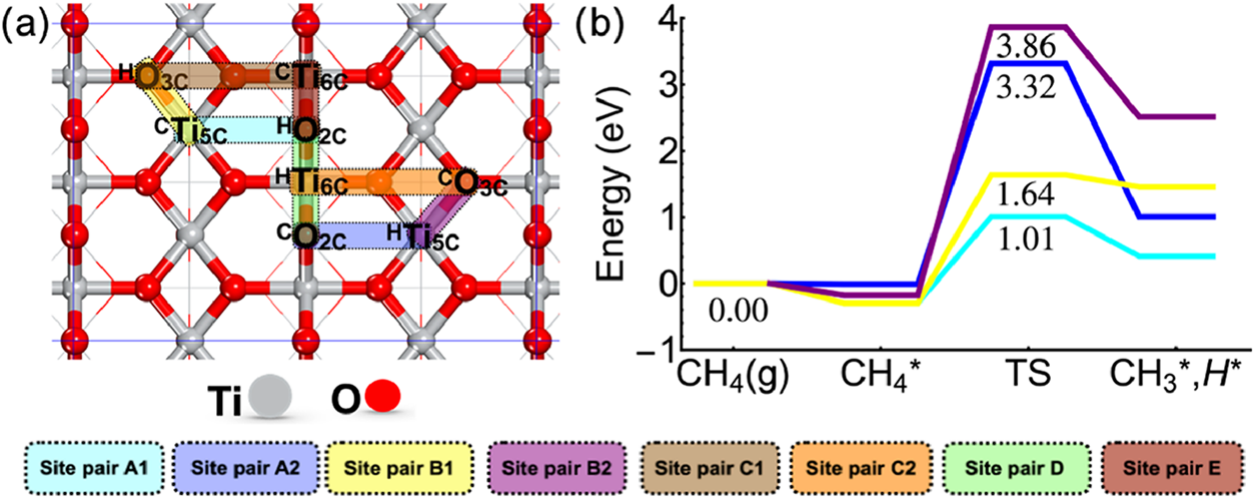
(a) Top view of different metal–oxygen adsorption site pairs on a rutile TiO_2_ (110) surface. (b) Methane C–H bond activation energy profile on corresponding site pairs (color coded).

#### 3.1.2 Dissociation of Two CH_4_ Molecules at a High Temperature

The Gibbs free energies for CH_4_ activation on the TiO_2_ (110) surface and the geometries of ISs, TSs, and FSs are depicted in Figure 2. At 1240 K, the entropy loss for CH_4_ adsorption was significant and resulted in endergonic CH_4_ adsorption of 0.76 and 1.05 eV for the first and second CH_4_, respectively. We noticed that the adsorption energy of the second CH_4_ molecule is 0.29 eV more endergonic than the adsorption energy of the first CH_4_ molecule owing to the steric hindrance that stems from the adjacent surface methyl and hydrogen species, making this configuration less stable. The activation energy barrier for the dissociation of the second CH_4_ molecule was found to be 0.31 eV higher than that of the first CH_4_ dissociation (1.42 eV). Huang et al. studied dual-metal-based nitrogen-doped graphene (Co_2_–N–C) and reported activation barriers of 2.25 and 2.46 eV for the first and second CH_4_ activation at 1200 K.^13^ Additionally, the activation of CH_4_ on molybdenum carbide exhibited a C–H bond activation barrier of 1.60 eV at 1023.15 K.^52^ The dissociation of the second CH_4_ yielded surface-bound Ti-methyl and hydroxyl species of carbanionic and protic characters, respectively. Overall, the dissociation of two CH_4_ molecules resulted in two pairs of H* and CH_3_^*^ species. The CH_3_^*^ can couple to form C_2_H_6_ or undergo further dehydrogenation to form CH_2_^*^ species, which can further couple to form C_2_H_4_. In the following sections, we discuss the different pathways of CC coupling to form C_2_H_6_ and C_2_H_4_.

**Figure 2:**
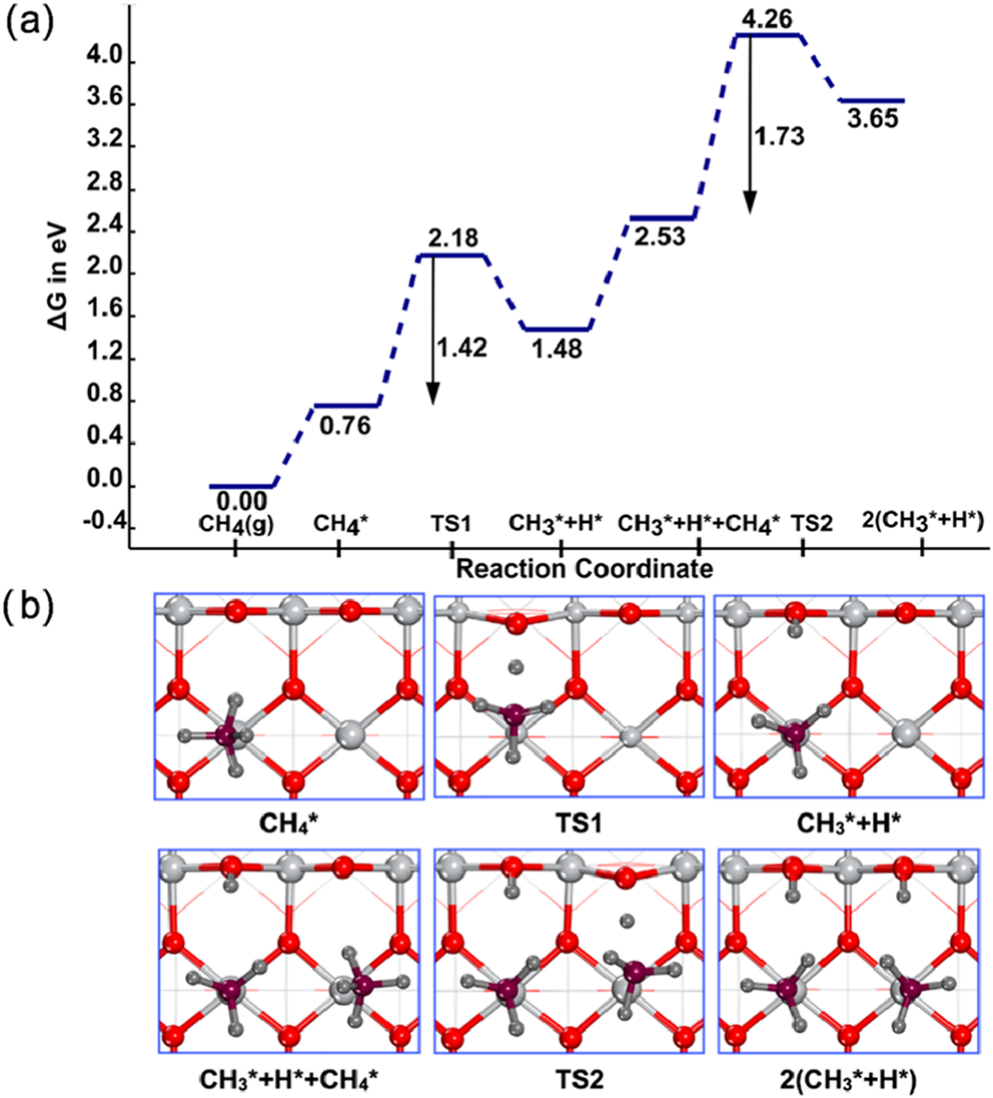
Dissociation of two CH_4_ molecules on the rutile TiO_2_(110) surface. (a) Free energy profiles of CH_4_ dissociation at 1240 K and (b) geometric structures of the ISs, TSs, and FSs involved in panel (a). Key: Ti: light gray; O: red; C: purple; H: dark gray.

### 3.2 Thermocatalytic C–C Coupling to **C**_**2**_ **H**_**6**_ and **C**_**2**_ **H**_**4**_ on TiO_2_

#### 3.2.1 Formation of C_2_H_6_

We identified three different CC coupling pathways for the formation of C_2_H_6_ on rutile TiO_2_. The free energy profiles for *C*_2_ *H*_6_ formation along with the geometries of related intermediates and TSs are shown in Figures 3 and S3. It is important to note that some elementary steps involved in the C_2_H_6_ formation reaction network require high activation energy barriers. However, our main goal is to explore the energetics for all possible elementary reactions and provide mechanistic insights into the catalytic activation of CH_4_ and its conversion to C_2_ hydrocarbons on rutile oxides. Pathway 1 is initiated by the direct formation of molecular H_2_ through the abstraction of a hydride from the methyl species by surface H* adsorbed on O_2c_, with an activation energy barrier of 2.72 eV. Although the barrier of H_2_ formation is high, the subsequent CC coupling (CH_3_^*^/CH_2_^*^) to form CH_3_CH_2_^*^ was found to be less energetically demanding, with *G*_a_ of 0.34 eV. The reaction energy of the CH_3_^*^/CH_2_^*^ coupling step was highly exothermic, with Δ*G* of −3.45 eV. The next step was the diffusion of H* from a O_2c_ atom to an adjacent O_2c_, which was found to be necessary for the hydrogenation of CH_3_CH_2_^*^ to C_2_H_6_. The energy barriers for H* diffusion and C_2_H_6_ formation were 1.45 and 2.33 eV, respectively. A similar mechanism was observed on dual-metal site catalysts on nitrogen-doped graphene where the H* diffuses or flips from one side to another side on graphene.^13^ Pathway 2, on the other side, starts with the diffusion of one CH_3_^*^ species to a neighboring O_3c_ site (*G*_a_= 2.84 eV), followed by the molecular H_2_ formation (*G*_a_ = 1.99 eV) via recombination of two surface-bound hydrogen atoms, and finally CH_3_^*^/CH_3(d)_^*^ coupling to form C_2_H_6_ (*G*_a_ = 1.76 eV), where d indicates the diffused state of the methyl species. All attempts to form H_2_ molecules without methyl diffusion failed. In these calculations, the guess geometry for the FS (where CH_3_^*^ species are bonded to Ti_5c_) converged to a state where one methyl species is bound to O_3c_ site. The diffusion of CH_3_^*^ to a neighboring O_3c_ increases the stability of CH_3_^*^ species by forming a strong O–CH_3_^*^ bond. While the hydrogen atoms remain protonic, the diffusion of CH_3_^*^ leads to charge redistribution on the surface, altering the electronic environment (Bader charge analysis; Table S2). This redistribution, combined with the polarization of the CH_3_ group (C10 becoming electron-deficient), likely facilitates H_2_ formation via two protons (H^+^) recombining. The projected density of state (Figure S6) indicates that the peak responsible for Ti_5c_–C bond shifts from −0.3 eV to −2.6 eV. Also, the new peak appears at −8.5 eV corresponding to O_3c_–C bond. This redistribution of the electron density may weaken nearby O–H bonds, further promoting proton recombination and H_2_ formation. Jenkins et al.^53^ reported that the diffusion of CH_3_^*^ from Pd sites to O sites on the PdO (110) surface enhances its stability due to the strongly covalent bonding between O and CH_3_^*^. Hence, the methyl diffusion step was necessary for the H_2_ formation reaction. Finally, pathway 3 involves direct coupling of two CH_3_^*^ species to form C_2_H_6_ in the presence of two surface-bound H* species, with a free energy activation barrier of 2.93 eV. Then, H_2_ molecule was formed via the recombination of dissociated hydrogen species, with an activation energy barrier of 2.34 eV. It is worth noting that at high temperatures such as 1240 K, numerous reactions, including carbon deposition, might occur and reduce the catalyst activity.^54^ According to the literature, the supported noble metals such as Rh, Ru, Pt, Ir, and Pd with appropriate support catalysts have high activity and strong carbon deposition resistance.^55,56^ Feng and co-workers used TiO_2_ as an additive to provide a physical barrier to hydrocarbon adsorption and decomposition on the catalyst surface and enhance the anticoking properties and catalytic activity of Ni–Ti/Al_2_O_3_ compared to Ni/Al_2_O_3_.^57^

**Figure 3:**
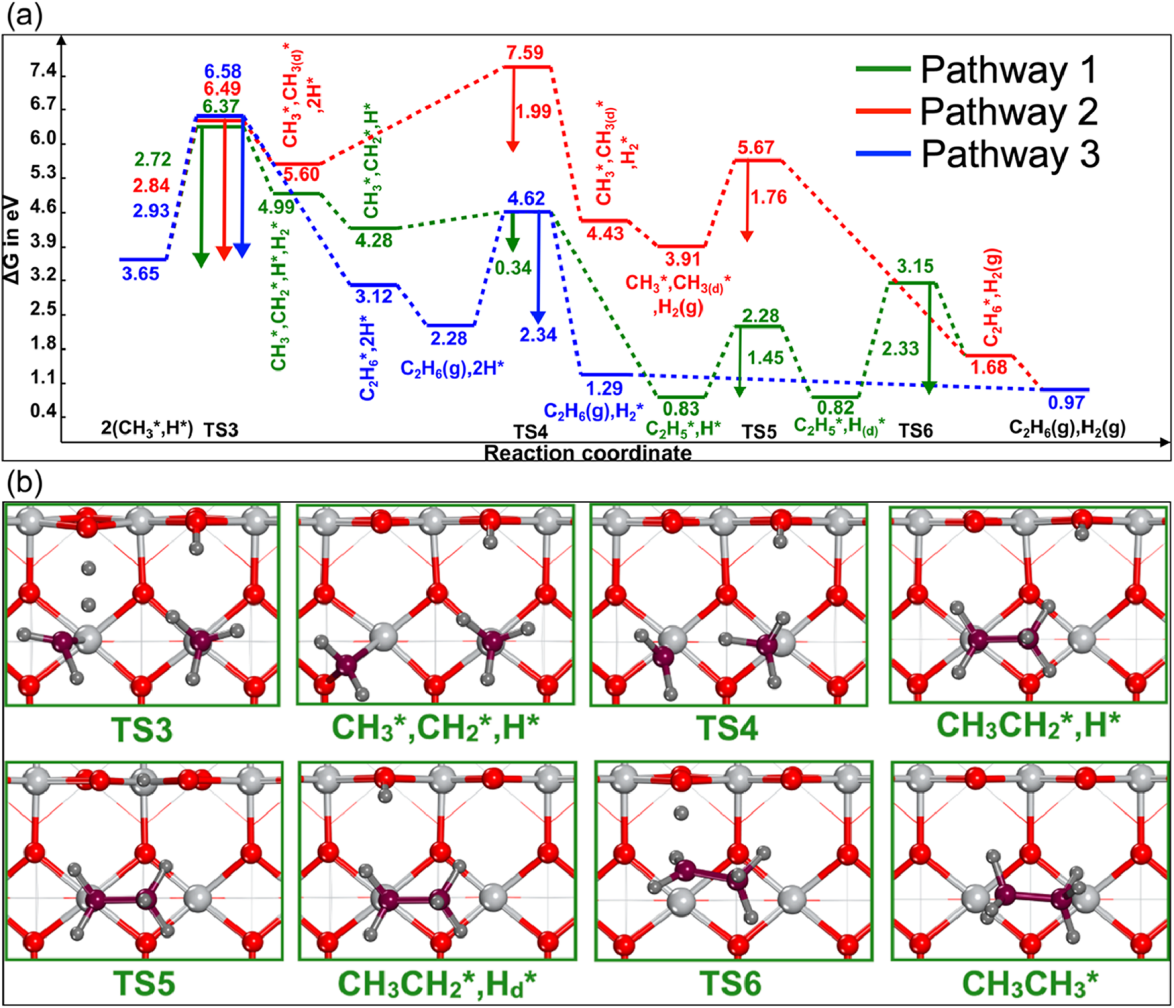
(a) Free energy profiles of C_2_H_6_ formation on the TiO_2_ (110) surface at 1240 K and (b) selected corresponding geometric structures of TSs and intermediates for the most kinetically preferred pathway 1. Selected geometries for pathways 2 and 3 are reported in Figure S3. Superscripts d indicate the diffusion of the species on the surface. Key: Ti: light gray; O: red; C: purple; H: dark gray.

In general, direct CC coupling (pathway 3, TS3) of two CH_3_–Ti_5c_ species is energetically demanding, whereas the diffusion of one of the CH_3_^*^ species to a neighboring O_3c_ site facilitates the CC coupling reaction (pathway 2, TS5) via decreasing the activation barrier significantly by 1.17 eV. All of the CC coupling reactions (CH_3_/CH_3_, CH_3_/CH_3(d)_, and CH_3_/CH_2_) were found to be exothermic, with reaction energies of −3.45, −2.23, and −0.53 eV for pathways 1, 2, and 3, respectively. The coupling of two surface CH_3_ (pathway 2 and 3) was more energetically demanding than CH_3_/CH_2_ (pathway 1), even at an elevated temperature of 1240 K. Considering the energy span between the highest-lying TS and lowest-lying intermediate,^58^ the pathway involving CH_3_/CH_2_ coupling (pathway 1) was found to be kinetically preferred, with an energy span value of 6.37 eV, compared to energy span values of 7.59 and 6.49 eV for pathways 2 and 3, respectively.

#### 3.2.2 Formation of C_2_H_4_

We identified three different pathways for C_2_H_4_ formation, as shown in Figure 4. The selected geometries of the kinetically favored pathway (pathway 2), which is the continuation of pathway 1 in the case of C_2_H_6_ formation, are shown in Figure 4, whereas pathways 1 and 3 are outlined in Figure S4. C_2_H_4_ formation pathways involve (1) dehydrogenation of CH_3_^*^ to CH_2_^*^ followed by CC coupling of two methylene groups, (2) dehydrogenation of CH_3_CH_2_^*^ followed by H_2_ formation, and (3) concerted dehydrogenation of C_2_H_6_ (simultaneous activation of two C–H bonds)^20^ followed by H_2_ formation. For all the pathways, the formation of the first molecular H_2_ from two initial dissociated surface H* was necessary to regenerate the bridge oxygen (O_2c_) active sites, which facilitated further dehydrogenation of CH_3_^*^ to CH_2_^*^. Our DFT calculations revealed that CC coupling of two CH_3_^*^ (pathway 3) is more energetically demanding, by 0.44 eV, than dehydrogenation of CH_3_^*^ to CH_2_^*^ (pathway 1). We noticed that the C–H bond activation of CH_3_^*^ is more facile than the C–H bond scission of CH_4_, by 0.10 and 0.41 eV for the activation of first and second CH_4_ molecules, respectively. In the dehydrogenation reaction of CH_3(d)_^*^ (bonded to O_3c_) in pathway 1, the CH_2_^*^ species was stabilized by both the O_3c_ and Ti_5c_ atoms, resulting in a lower activation energy barrier (1.32 eV) compared to the dissociation of CH_4_ (1.42 eV). However, the dehydrogenation of the second CH_3_^*^ species (bonded to Ti_5c_) exhibited a higher activation energy barrier of 3.55 eV. The higher activation barrier can be attributed to the steric hindrance from dissociated CH_2_^*^ and H* on the surface. This steric hindrance forces the first dissociated H* to reorient itself away from the first dissociated CH_2_^*^, as shown in the CH_3_^*^, CH_2_^*^, H*, and 2­(CH_2_^*^, H*) states of Figure S4. Additionally, the C–H bond activation of the second CH_3_^*^ resulted in simultaneous bond dissociation of CH_3_–Ti_5c_ and formation of two bonds: (i) H–O_2c_ and (ii) CH_2_–Ti_5c_, where Ti_5c_ is another neighboring Ti atom that participates in the stabilization of the first formed CH_2_^*^ species. Similar TSs involving simultaneous bond dissociation and generation have also been located and observed by *ab initio* molecular dynamics simulations and DFT calculations.^13,59^ During the coupling of two methyl groups on the dual-metal sites on graphene, dehydrogenation of one CH_3_^*^ takes place simultaneously, resulting in the final intermediate CH_3_CH_2_^*^ adsorbed on the top site of one metal atom and H* adsorbed at the bridge site of dual-metal sites^13^ at 1200 K. Then, the subsequent step involves the hydrogenation of CH_3_CH_2_^*^ to form ethane.

**Figure 4:**
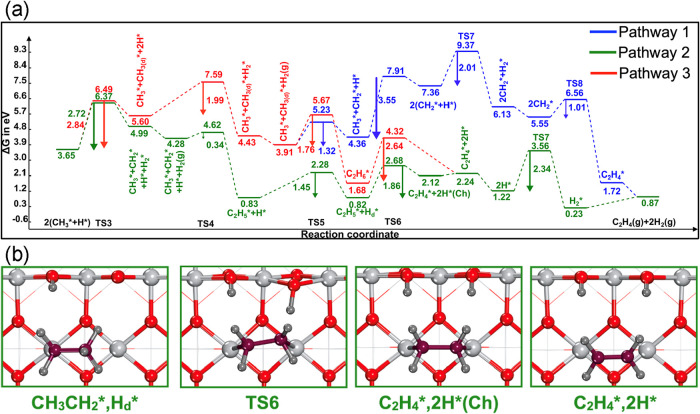
(a) Free energy profiles of C_2_H_4_ formation on the rutile TiO_2_ (110) surface at 1240 K. (b) Selected corresponding geometric structures of TSs and intermediates of kinetically favored pathway 2. To simplify the labels of the intermediates and products, H_2_(g) produced is omitted. Here, subscript d indicates the diffusion of the species on the surface. Key: Ti: light gray; O: red; C: purple; H: dark gray.

In the next step of pathway 1, molecular H_2_ is formed via surface hydrogen recombination, followed by CH_2_^*^/CH_2_^*^ coupling to form C_2_H_4_^*^, with activation energy barriers of 2.01 and 1.01 eV, respectively. The CH_2_^*^/CH_2_^*^ coupling reaction was found to be highly exothermic, with Δ*G* of −3.83 eV. Similarly, CH_2_ self-coupling reactions to form C_2_H_4_ are favored both kinetically and thermodynamically over CH_3_/CH_3_ coupling on IrO_2_/TiO_2_,^35^ Mo_2_C, and MoC surfaces.^60,61^ Furthermore, pathway 2 involves the dehydrogenation of C_2_H_5_^*^ to form chemisorbed C_2_H_4_^*^ (Ch) and H* species, with an activation energy barrier of 1.86 eV in Figure 4b. However, this reaction was found to be highly endothermic, with a reaction energy of 1.30 eV. Our DFT calculations revealed that the chemisorbed C_2_H_4_^*^ (Ch) prefers to spontaneously physisorb due to entropic contributions at the elevated temperature of the simulation. In pathway 3, C_2_H_4_ was formed by simultaneous abstraction of two hydrogens of C_2_H_6_, with an activation barrier of 2.64 eV and Δ*G* of 0.56 eV. The formed C_2_H_4_^*^ was desorbed into the gas phase, with a release of energy of 1.02 eV. Then, the two surface-bound H* recombined to form molecular H_2_, with an activation energy barrier of 2.34 eV, leading to catalyst regeneration. Additionally, H_2_ formation was found to be exothermic, with a reaction energy of −0.99 eV. By comparing the energy span values of different C_2_H_4_ formation pathways, we noted that pathway 2 (dehydrogenation of surface-bound C_2_H_5_^*^) is kinetically more favored, with an energy span value of 6.37, compared to 9.37 and 7.59 eV for pathways 1 and 3, respectively.

As the CC coupling reactions are the most critical steps in NOCM, we summarize the calculated reaction Gibbs free energies and activation energy barriers for the four different CC coupling reactions involved in C_2_H_6_/C_2_H_4_ formation in Table 1, and the corresponding ISs, TSs, and FSs structures are depicted in Figure S5. The CC coupling reactions involved in C_2_H_6_ and C_2_H_4_ formation pathways were all exothermic, where the reaction energies varied from −3.83 to −0.53 eV. Among all CC coupling reactions [CH_3_/CH_3_, CH_3_/CH_3(d)_, CH_3_/CH_2_, and CH_2_/CH_2_], CH_3_/CH_2_ coupling to C_2_H_5_^*^ was found to be relatively favored both kinetically and thermodynamically. Interestingly, we observed that dehydrogenation of C_2_H_5_^*^ to C_2_H_4_ is energetically more favored than further hydrogenation to form C_2_H_6_ (with activation energy barriers of 1.86 and 2.33 eV for the dehydrogenation and hydrogenation reactions, respectively).

**Table 1:** Gibbs Activation Free Energy Barriers (*G*_A_) and Reaction Energies (*G*_R_) of CC Coupling Reactions at 1240 K for Different NOCM Pathways on TiO_2_ ^a^

elementary reactions	*G*_A_	*G*_R_
${\mathrm{CH}}_{3}+{\mathrm{CH}}_{2}+H\to {\mathrm{CH}}_{3}{\mathrm{CH}}_{2}+H{\left(\mathrm{ane}1/\mathrm{ene}2\right)}^{*}$	0.34	–3.45
${\mathrm{CH}}_{3}+{\mathrm{CH}}_{3}\left(d\right)\to C_{2}H_{6}\left(\mathrm{ane}2/\mathrm{ene}3\right)$	1.76	–2.23
$2{\mathrm{CH}}_{3}+2H\to C_{2}H_{6}+2H\left(\mathrm{ane}3\right)$	2.93	–0.53
$2{\mathrm{CH}}_{2}\to C_{2}H_{4}\left(\mathrm{ene}1\right)$	1.01	–3.83

a All free energies are reported in eV. * ane → ethane pathway and ene → ethylene pathway.

Although the DFT calculations showed that thermocatalytic NOCM is inactive on pristine TiO_2_, even at high temperatures, the detailed mechanistic insights provided in this work can be transferred to other rutile oxides such as IrO_2_, PtO_2_, etc., due to the similar structural properties, including Lewis acidity and reducibility of the rutile oxides. However, several studies demonstrated that defect-rich TiO_2_ including oxygen vacancies^62^ and transition-metal-doped^63^ TiO_2_ exhibit enhanced photocatalytic activity toward the dissociative adsorption of O_2_, H_2_, CO, and H_2_O molecules. However, there is no experimental or theoretical work in the literature solely based on the thermocatalytic behavior (activity or inertness) of pristine rutile TiO_2_ and the conversion of CH_4_ to C_2_ hydrocarbons. In this context, we focused on pristine TiO_2_ to explore different pathways of the NOCM to C_2_ hydrocarbons rather than defect-rich rutile. Our DFT results and mechanistic pathways provide an excellent guideline for future studies to design effective rutile-type metal-oxide dehydrogenation catalysts.

## 4 Conclusions

In this work, we applied periodic DFT calculations to investigate the nonoxidative coupling of methane to ethane and ethylene on the rutile TiO_2_ (110) surface at 1240 K. Our DFT calculations revealed that the diffusion of one methyl species to the neighbor O_3c_ followed by molecular hydrogen formation facilitates CH_3_/CH_3_ coupling. Moreover, we found that CH_3_/CH_2_ coupling is kinetically and thermodynamically preferred to direct coupling of CH_3_/CH_3_ and CH_2_/CH_2_. Interestingly, dehydrogenation of surface ethyl species to ethylene was more energetically favored than hydrogenation to ethane. These results provide detailed mechanistic insights into the NOCM on rutile TiO_2_, demonstrating its inertness in activating C–H bonds and converting methane to higher hydrocarbons. Toward rational development of active and selective catalysts to activate and convert methane to valuable C_2+_ hydrocarbons, our study suggests that alternative to pristine TiO_2_ catalytic sites need to be explored by modification of TiO_2_ through surface doping or O-vacancy formation.

## Supplementary Material



## Notes

Optimizing *U* values for Ti-3d orbitals, reaction coordinates of CH bond activation, geometries of the TSs and intermediates for the (ethane and ethylene) formation, and C–C coupling (PDF)
