# Supplementary material for: Thermocatalytic Behavior of TiO2 as a Dehydrogenation Catalyst: A Case Study of Methane Activation and Nonoxidative Coupling
Source: ACS Omega. 2026 Jan 20;11(4):6285–92. doi: 10.1021/acsomega.5c10988 (PMC12878774; doi:10.1021/acsomega.5c10988)
Supplement: Supplementary file 1 [file ao5c10988_si_001.pdf]

Supporting Information

Thermocatalytic Behavior of TiO_2 as a Dehydrogenation Catalyst: A Case Study of Methane Activation and Nonoxidative Coupling

Juganta K. Roy[1,2*], Mona Abdelgaid[1], and Giannis Mpourmpakis[1,3]

[1] Department of Chemical and Petroleum Engineering, University of Pittsburgh, Pittsburgh, Pennsylvania 15261, United States

[2] Department of Chemistry and Physics, West Texas A&M University, Canyon, Texas 79016, United States

[3] School of Chemical Engineering, National Technical University of Athens (NTUA), Athens, GR-15780, Greece

SI	Name of the Figures/Tables	Page No
Figure S1	Optimization of U values of Ti-3d orbital for DFT+U calculations	S2
Figure S2	Reaction coordinates of CH bond activation on different adsorption sites of TiO2(110) surface.	S3
Figure S3	Geometries of the TSs and intermediates for the formation of ethane via pathway 2 and pathway 3.	S4
Figure S4	Geometries of the TSs and intermediates for the formation of ethylene via pathway 1 and pathway 3.	S5
Figure S5	Geometries of the TSs and intermediates different C-C coupling reactions on the TiO_2 (110) surface.	S6
Figure S6	Projected density of states (PDOS) of the intermediates of before and after diffusion of CH_3 group in pathway 2 (ethane formation).	S7
Table S1	CH_4 adsorption energies, dissociative reaction energies, and activation energy on different sites of rutile (110) surface	S8
Table S2	Bader charge analysis of the intermediates during the methyl diffusion in pathway 2 of ethane formation.	S9

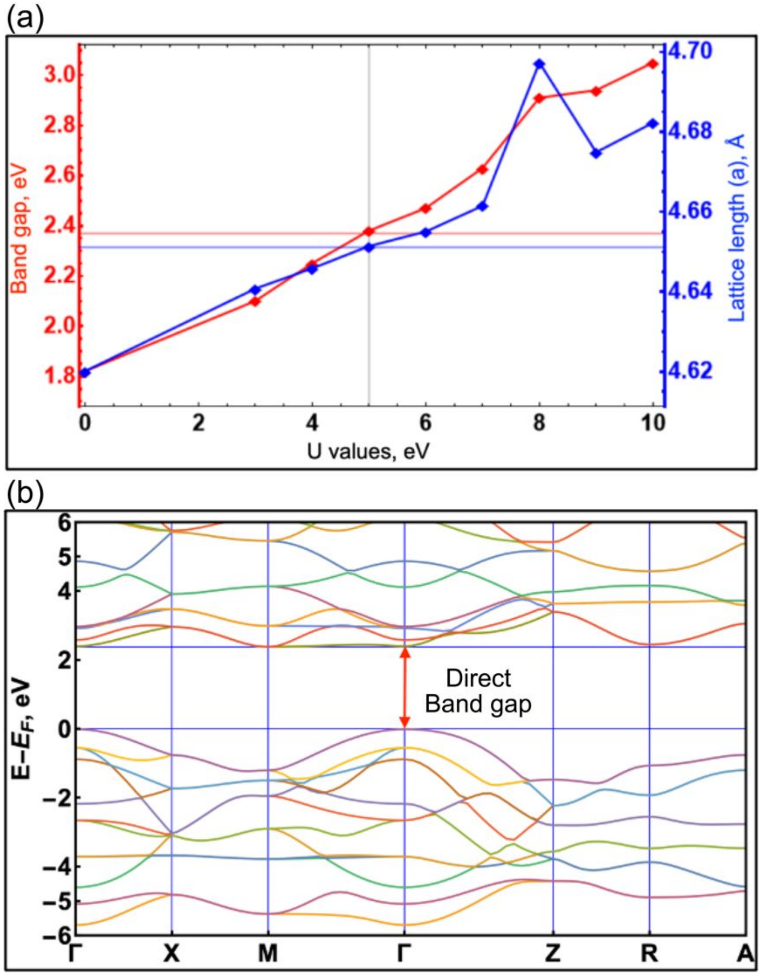

Figure S1. Optimization of U values for Ti-3*d* orbital. Experimental values form the ref [1]. (a) Variation of lattice constant (blue line) and band gap (red line) with respect to different U values, and (b) Band structure of rutile unit cell at Ti-3*d* (U=5.0).

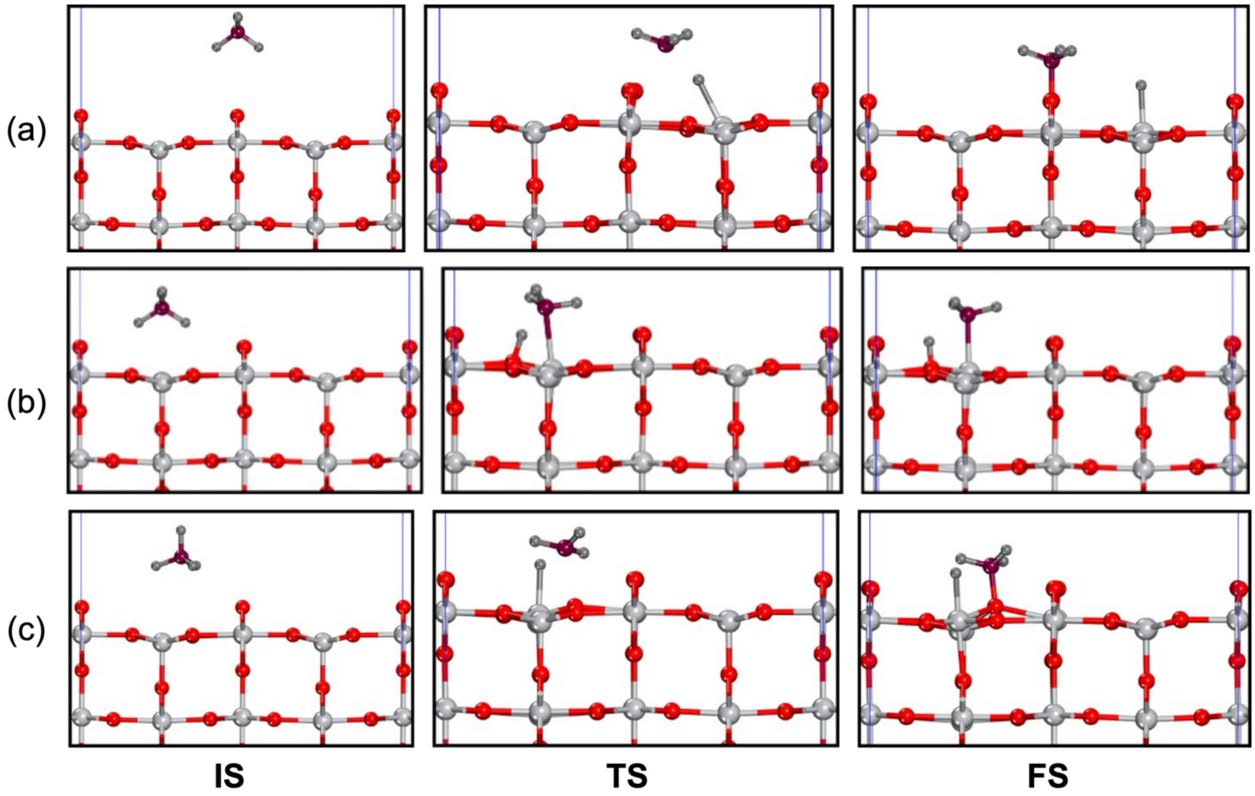

Figure S2. Initial state (IS), transition state (TS), and final state (FS) for the methane's C-H bond activation on different adsorption sites (a) siteA2 ($^{C}O_{2c} - {}^{H}Ti_{6c}$), (b) siteB1 ($^{C}Ti_{5c} - {}^{H}O_{3c}$), and (c) siteB2 ($^{C}O_{3c} - {}^{H}Ti_{5c}$) on the TiO_2 (110) surface.

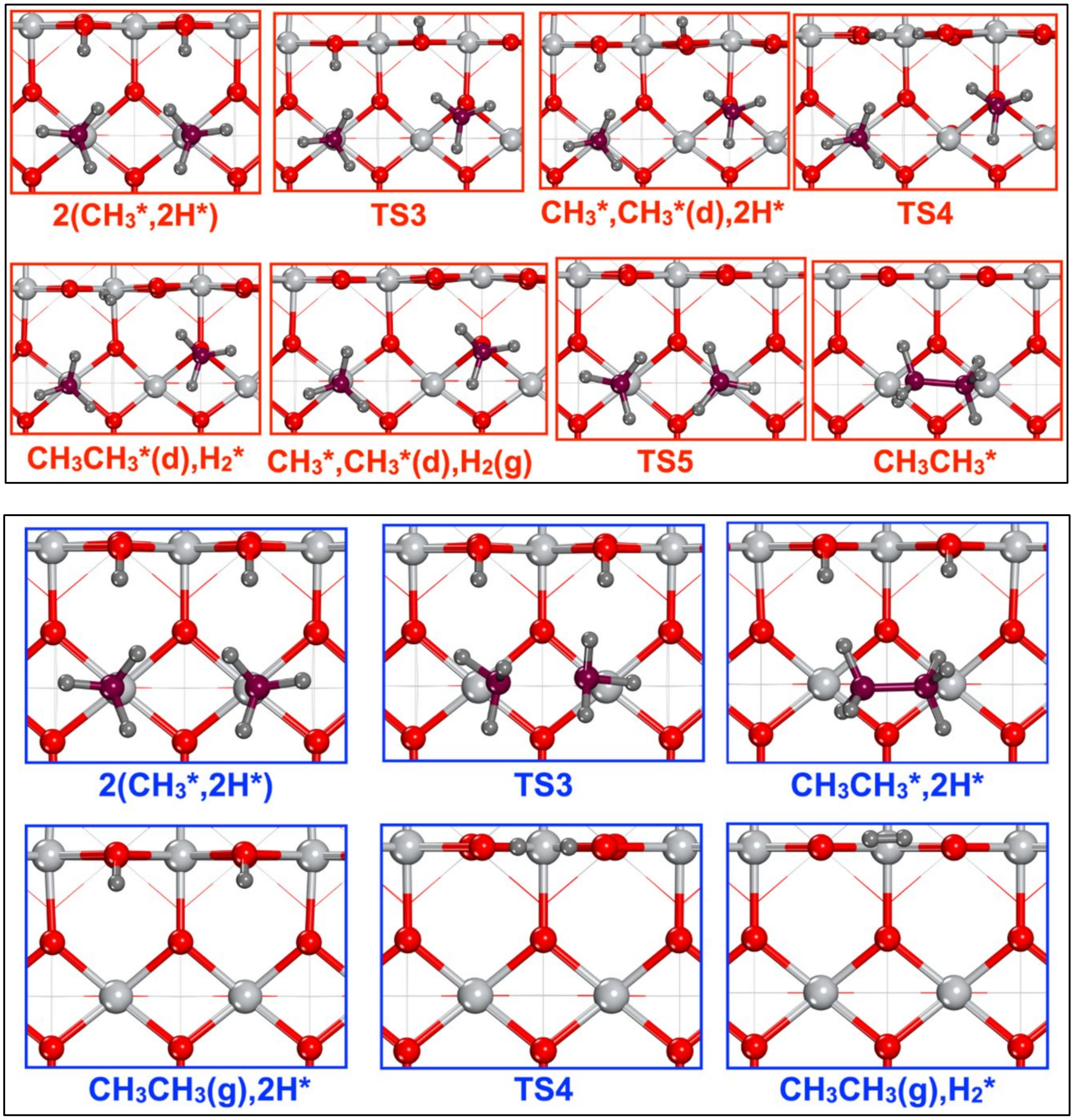

Figure S3. Geometries of the TSs and intermediates for the formation of ethane via pathway 2 (top) and pathway 3 (bottom).

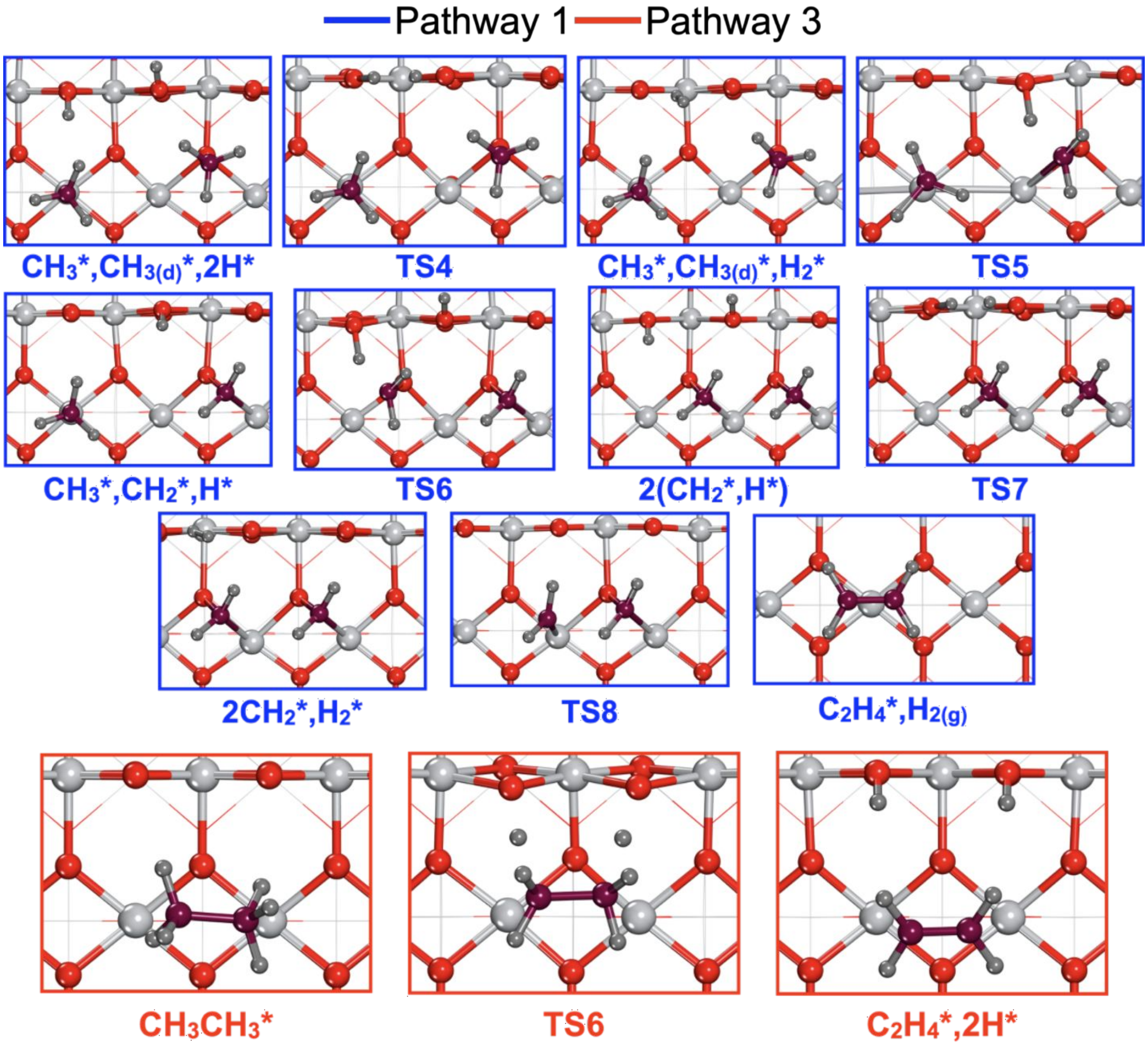

Figure S4. Geometries of the TSs and intermediates for the formation of ethylene via pathway 1 (blue) and pathway 3 (red).

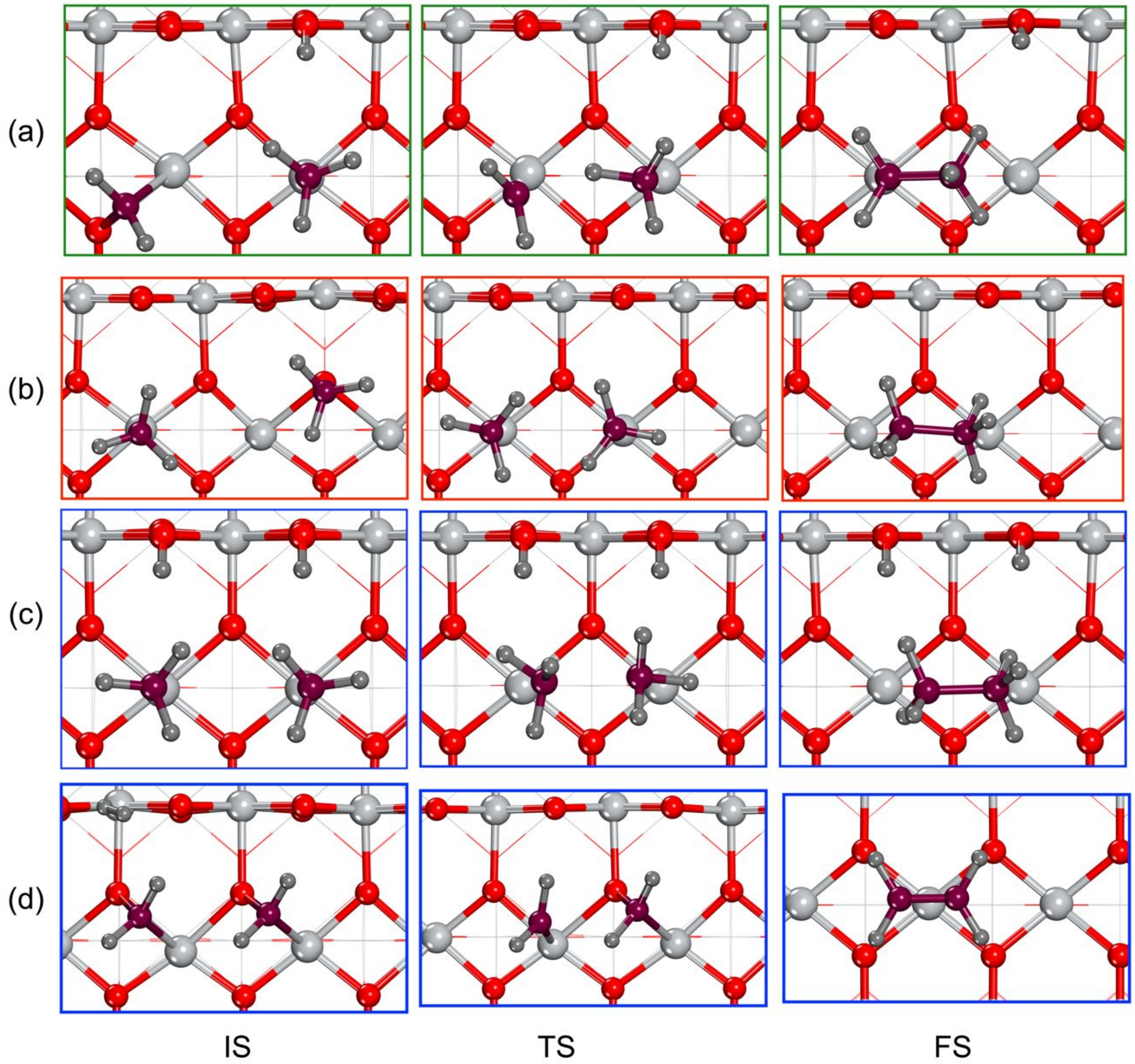

Figure S5. Geometries of the TSs and intermediates for CC coupling reactions of (a) CH_3/CH_2 in pathway 1, (b) CH_3/CH_3 in pathway 2, (c) CH_3/CH_3 in pathway 3, and (d) CH_2/CH_2 pathway 1 on the TiO_2 (110) surface.

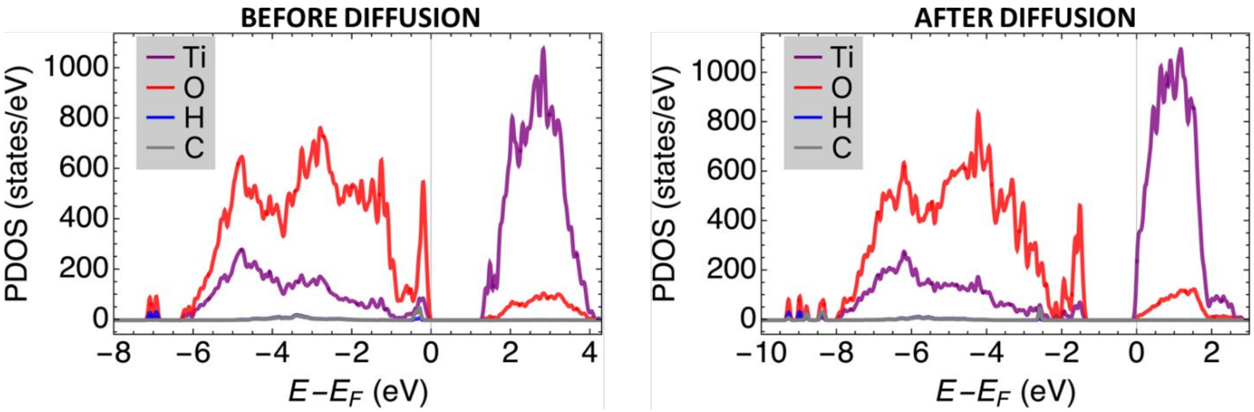

Figure S6. Projected density of states (PDOS) of the intermediates of before and after diffusion of CH_3 group in pathway 2 (ethane formation).

Table S1. CH_4 adsorption energies, dissociative reaction energies, and activation energy of the different sites of rutile (110) surface. All the energies are electronic energy and in eV.

Adsorption modes	Adsorption Energy, eV	Activation Energy, eV	Reaction Energy, eV
SiteA1	-0.30	1.01	0.41
SiteA2	-0.02	3.32	1.01
SiteB1	-0.30	1.64	1.45
SiteB2	-0.18	3.86	2.51
SiteC1	-0.02	-	3.91
SiteC2	-0.18	-	4.32
SiteD	-0.03	-	0.86
SiteE	-0.03	-	2.78
Frad	-0.03	-	1.88

Table S2. Bader charge analysis of the intermediates during the methyl diffusion in pathway 2 of ethane formation.

atom	before diffusion	after diffusion
O2C	-1.18	-1.11
H7	0.49	0.48
O2C	-1.17	1.64
H2	0.51	0.52
C9	-0.004	-0.28
C10	-0.006	0.51
O3C	-1.15	-0.92

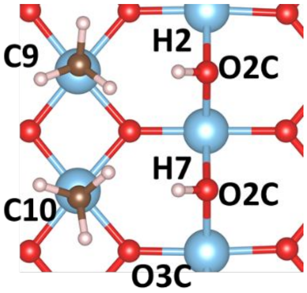

References:

[1] Diebold U. The surface science of titanium dioxide. Surf Sci Rep 2003;48:53–229. https://doi.org/10.1016/S0167-5729(02)00100-0.
